# Schizophrenia, amphetamine-induced sensitized state and acute amphetamine exposure all show a common alteration: increased dopamine D2 receptor dimerization

**DOI:** 10.1186/1756-6606-3-25

**Published:** 2010-09-02

**Authors:** Min Wang, Lin Pei, Paul J Fletcher, Shitij Kapur, Philip Seeman, Fang Liu

**Affiliations:** 1 Department of Neuroscience, Centre for Addiction and Mental Health, Toronto, Ontario, M5T 1R8, Canada; 2 Institute of Psychiatry, University of London, London, SE5 8AF, UK; 3 Department of Psychology, University of Toronto, Ontario M5S 3GS, Canada; 4 Department of Psychiatry, University of Toronto, Ontario, M5T 1R8, Canada; 5 Department of Pharmacology and Toxicology, University of Toronto, Ontario, M5S 1A8, Canada; 6 Department of Neuroscience, Centre for Addiction and Mental Health, Clarke Division, 250 College Street, Toronto, Ontario M5T 1R8, Canada

## Abstract

**Background:**

All antipsychotics work via dopamine D2 receptors (D2Rs), suggesting a critical role for D2Rs in psychosis; however, there is little evidence for a change in receptor number or pharmacological nature of D2Rs. Recent data suggest that D2Rs form dimers in-vitro and in-vivo, and we hypothesized that schizophrenia, as well as preclinical models of schizophrenia, would demonstrate altered dimerization of D2Rs, even though the overall number of D2Rs was unaltered.

**Methods:**

We measured the expression of D2Rs dimers and monomers in patients with schizophrenia using Western blots, and then in striatal tissue from rats exhibiting the amphetamine-induced sensitized state (AISS). We further examined the interaction between D2Rs and the dopamine transporter (DAT) by co-immunoprecipitation, and measured the expression of dopamine D2^High ^receptors with ligand binding assays in rat striatum slices with or without acute amphetamine pre-treatment.

**Results:**

We observed significantly enhanced expression of D2Rs dimers (277.7 ± 33.6%) and decreased expression of D2Rs monomers in post-mortem striatal tissue of schizophrenia patients. We found that amphetamine facilitated D2Rs dimerization in both the striatum of AISS rats and in rat striatal neurons. Furthermore, amphetamine-induced D2Rs dimerization may be associated with the D2R-DAT protein-protein interaction as an interfering peptide that disrupts the D2R-DAT coupling, blocked amphetamine-induced up-regulation of D2Rs dimerization.

**Conclusions:**

Given the fact that amphetamine induces psychosis and that the AISS rat is a widely accepted animal model of psychosis, our data suggest that D2R dimerization may be important in the pathophysiology of schizophrenia and may be a promising new target for novel antipsychotic drugs.

## Background

Schizophrenia is a chronic mental illness, characterized by episodes of psychotic symptoms that usually emerge in early adulthood, last a lifetime, and destroy the mental and interpersonal faculties most valued in human society. The fact that all current antipsychotic drugs exert their effect through the blockade of D2R emphasizes the critical role of hyper-dopaminergic neurotransmission through D2R signaling in the pathophysiology of schizophrenia [1]. Yet, studies are conflicting about the role of D2R in the illness. While postmortem studies have demonstrated an increase in the overall number of D2R, this is confounded by the fact that this increase could be a compensatory response to D2R blockade rather than a diagnostic feature of schizophrenia [2]. On the other hand, PET imaging studies show unequivocal support for a change in D2R [3]. Several recent PET imaging studies show a well-replicated increase in dopamine release in patients with schizophrenia, and even during the prodromal phase [4]. Thus, it remains unclear what, if any, the direct role D2R play in the pathophysiology of schizophrenia.

Most previous studies have measured D2R using receptor radioactive ligand binding assay, PET imaging or Western blot analysis. These techniques provide a good index of overall binding sites, but do not provide any insight into molecular aspects of receptor functions, including their potential configurations with other receptors. Several GPCRs exist in homo- or hetero-dimers to allow multiple signal integrations [5]. A number of studies have focused on the role of dimerization in regulating the function of GPCRs [5]. Indeed, dimerization of GPCRs may alter pharmacological properties and signaling transduction, leading to significant effects on cellular physiology as well as disease pathologies. For example, agonist occupancy of one of the two ligand binding site of the β_2_-adrenoceptor dimers is sufficient to cause internalization of the dimers. However, both binding sites have to be occupied by the antagonist to prevent agonist-mediated internalization [6]. Furthermore, receptor hetero-dimerization makes it possible to modulate one receptor using ligands targeting the other receptor [7-9]. These unique properties of receptor oligomerization have made them novel targets for the development of novel drugs [10].

Previous studies indicate that D2R are expressed as monomers and dimers in cell lines and in mammalian brain tissue [11-14]. Furthermore, D2R have also been reported as dimers in a variety of neurological disease such as Alzheimer's, Parkinson's and Huntington's disease [15,16]. As a result, interest has focused on the potential pathophysiological role of D2R dimerization in disease. Although pharmacological studies have suggested that D2R dimers and monomers have differential affinity for specific dopamine receptor ligands that may subsequently affect dopamine release [17,18], the functional role of the D2R dimerization and the molecular mechanisms involved are not well understood.

Thus, the purpose of this study was to examine the status of D2R dimers and monomers in schizophrenia. We started with analysis of postmortem striatal sections from schizophrenia patients and observed a significant increase in D2R dimers at the expense of monomers. To examine if this pathology was observed in preclinical models of schizophrenia that involve a hyper-dopaminergic state, we examined the status of D2R dimers in rats exhibiting an amphetamine-induced sensitized state (AISS). A similar increase in D2R dimerization was observed. To further study the molecular basis for this effect (and to link the alteration in D2 receptors to the human observation of alteration in presynaptic dopamine release) we examined the status of D2R as well as their link to the dopamine transporter (DAT) in cultured striatal neurons, with or without acute amphetamine (AMPH) exposure.

## Materials and methods

### Human postmortem brain tissue

Formalin-fixed paraffin-embedded human postmortem striatum sections (10 μm-thick on glass slides) were donated by the Stanley Foundation Neuropathology Consortium [19]. Subjects were divided into four groups, including bipolar (BD), major depressive disorder (MDD), schizophrenia (SZ), and non-neurological/non-psychiatric controls (n = 15 per group). Subjects were matched for age, gender, postmortem interval (PMI), pH, and mRNA quality. Demographic and medical information such as drug abuse history and psychotropic treatments were provided by the Stanley Foundation Neuropathology Consortium. Diagnoses were retrospectively established by two senior psychiatrists using DSM-IV criteria. All experiments were performed blinded to the diagnosis of each subject.

### Animals

Adult male Sprague-Dawley rats weighing 250-275 g were procured from Charles River Laboratories, Montreal, Canada. Animals were maintained on a 12-h light/dark cycle and housed two per cage with continuous access to food and water. The animals were allowed to acclimatize to the vivarium for a minimum of 5 days before being used for experimentation. All experimental protocols were approved by the CAMH animal care committee.

### Chronic haloperidol treatment

Chronic haloperidol treatment was performed as previously described [20]. Briefly, groups of 12 rats each were randomly assigned to receive one of the following treatments: 0.25 mg/kg/day of haloperidol (McNeil Pharmaceuticals, Spring House, PA) or vehicle via Alzet osmotic mini-pumps (Alzet model 2ML4, Durect Corporation, Cupertino, CA) for a total of 2 weeks, to achieve continuous clinical occupancy.

### Amphetamine-induced sensitized state

Amphetamine (Tocris Bioscience, Ellisville, MS) were given three times a week (Monday, Wednesday and Friday) for 5 weeks via intraperitoneal injection (IP). During week 1, amphetamine treated animals received a dose of 1 mg/kg (from salt); with the dose increasing by 1 mg/kg each week so that the dose in the final week was 5 mg/kg. Control animals received saline. All injections were administered in a 1 ml/kg volume.

### Acute striatal slices

Acute striatal slices (350 μm-thick) were prepared from Sprague-Dawley rats using a McIlwain tissue chopper (Mickle Laboratory Engineering, Gomshall, United Kingdom). Rat striata were dissected out and left for 5 min in ice-cold artificial cerebrospinal fluid (aCSF) containing 126 mM NaCl, 2.5 mM KCl, 1 mM MgCl_2_, 1 mM CaCl_2_, 1.25 mM KH_2_PO_4_, 26 mM NaHCO_3 _and 20 mM glucose, that was bubbled continuously with carbogen (95% O_2_/5% CO_2_) to adjust the pH to 7.4. Freshly cut slices were placed in an incubating chamber with carbogenated aCSF and recovered from stress at 37°C for 1 hour. Slices were then treated with 10 μM AMPH for 30 min. Slices were then harvested for Western blot analysis.

### Primary cultured striatal neurons

Primary cultures from striatum were prepared from fetal Wistar rats (embryonic day 17-19) on culture dishes as previously described [21]. The cultures were used for experiments 12-15 days after plating.

### HEK293T cell culture conditions and transfection

HEK293T cells were cultured in α-MEM (Invitrogen, Carlsbad, CA) supplemented with 10% fetal bovine serum (Invitrogen) and maintained in incubators at 37°C, 5% CO2. One day before transfection, cells were split onto poly-D-lysine coated plates. For lipofectamine2000 transfections, DAT: D2R cDNA ratio of 1:5 to maximize coexpression. Cells were utilized 2 days post transfection.

### Co-immunoprecipitation

Co-immunoprecipitation was performed as previously described [21,22]. Briefly, solubilized rat striatal extracts (500~700 μg) were incubated in RIPA buffer with primary antibody anti-D2 (Millipore, Billerica, MA) or rabbit IgG (1~2 μg, Sigma-Aldrich, St. Louis, MO) for 4 h at 4°C, followed by the addition of 20 μl of protein A/G agarose (Santa Cruz, Santa Cruz, CA) for 12 h. Pellets were washed, boiled for 5 min in SDS sample buffer and subjected to SDS-PAGE. 20~50 μg of tissue extracted protein was used as a positive control in each experiment. Anti-DAT (Santa Cruz) was applied as the immunoblotting antibody in the following western blot analyses.

### Western blot

Western blot analyses were performed as previously described [21,22]. Briefly, formalin-fixed paraffin-embedded frozen human postmortem striatal sections from the Stanley Consortium (scraped off the glass slides), rat striatum, HEK293T cells and cultured rat striatal neurons (~2 × 10^7^) were homogenized in RIPA buffer (50 mM Tris-Cl, pH 7.6, 150 mM NaCl, 2 mM EDTA, 1 mM PMSF plus 1% Igepal CA-630, 0.5% sodium deoxycholate, 1% Triton X-100) with a protease inhibitor cocktail (Sigma-Aldrich), and centrifuged at 4°C at 13,000 rpm for 10 min. Supernatant was extracted and protein concentrations were measured (Bio-Rad, Hercules, CA). Protein samples (50 μg) were boiled for 5 min in SDS sample buffer, and subjected to SDS-PAGE. Blots were blocked with 5% non-fat dried milk dissolved in TBST buffer (10 mM Tris, 150 mM NaCl, and 0.1% Tween 20) for 1 h at room temperature, washed three times with TBST buffer, incubated with the appropriate primary antibody [anti-D2 (polyclonal) or anti-α-tubulin (monoclonal, Sigma-Aldrich) diluted in 5% milk in TBST] overnight at 4°C. The blots were washed again with TBST buffer three times and then incubated with horseradish peroxidase-conjugated secondary antibody (diluted in 5% milk in TBST; Sigma-Aldrich) for 1.5 h at room temperate. The proteins were visualized with enhanced chemiluminescence reagents (GE Healthcare, Piscataway, NJ).

### Inhibition of [^3^H]-domperidone binding to D2R

The striatal tissue was homogenised (final concentration of 4 mg/ml) in a buffer containing 50 mM Tris-HCl (pH 7.4 at 20°C), 1 mM EDTA, 5 mM KCl, 1.5 mM CaCl_2_, 4 mM MgCl_2 _and 120 mM NaCl, using a Teflon-glass homogeniser with the piston rotating at 500 RPM and 10 up-and-down strokes of the glass container. Similar results were obtained with either washed or non-washed homogenates. Although it is known that approximately half the D2R can be lost upon washing the tissue [23], the homogenates were washed by centrifuging the homogenate at 10,000 × g for 10 min and discarding the supernatant; this procedure was repeated two more times. The final pellet was used.

The D2R in the striatal tissue were measured with [^3^H]-domperidone (2 nM final concentration; custom synthesized as [phenyl-^3^H(N)]-domperidone; 41.4 Ci/mmol; Moravek Radiochemicals Inc., Brea, CA) [24]. Each incubation tube (12 × 75 mm, glass) received, in the following order, 0.5 ml buffer, containing a range of dopamine concentrations, with or without a final concentration of 10 μM S-sulpiride (to define nonspecific D2R binding), 0.25 ml [^3^H]domperidone (generally 1.8 nM as the final concentration in the incubation tube), and 0.25 ml of tissue homogenate. Each concentration of dopamine was tested in duplicate. The tubes, containing a total volume of 1 ml, were incubated for 2 h at room temperature (20°C), after which the incubates were filtered, using a 12-well cell harvester (Titertek, Skatron, Lier, Norway) and buffer-presoaked glass fiber filter mats (Whatman GF/C). After filtering the incubates, the filter mat was rinsed with buffer for 15 s (7.5 ml buffer), and the filters were processed as detailed above. The specific binding of [^3^H]-domperidone was defined as total binding minus that in the presence of 10 μM S-sulpiride. Independent saturation of D2R, using a range of [^3^H]-domperidone concentrations, revealed a [^3^H]-domperidone dissociation constant (Kd) of 0.48 ± 0.08 nM (n = 6) for the rat homogenized striata.

### TAT peptides pre-treatment

The acute rat striatal slices were pre-treated with TAT-fused peptides (30 min, 10 μM) before amphetamine treatment. TAT-fused peptides were synthesized by GeneScript. Peptides are rendered cell permeant by fusing to the cell membrane transduction domain of the human immunodeficiency virus type 1 TAT protein (YGRKKRRQRRR), as previously described [25].

### Densitometry and statistical analysis

To quantify the bands obtained via Western blot analysis, we applied ImageJ software based analysis (http://rsb.info.nih.gov/ij/). The area under curve (AUC) of the specific signal was corrected for the AUC of the loading control (e.g. α-tubulin). All values are provided as means ± SEM. For comparisons between two groups, t-test (two-tailed) was performed. For comparisons of more than two groups, one-way ANOVA followed by SNK *post-hoc *analysis was performed. Unless otherwise noted, significance level was set at 0.05.

## Results

### Enhanced expression of D2R dimers in postmortem striatal sections from schizophrenia patients

**Figure 1:**
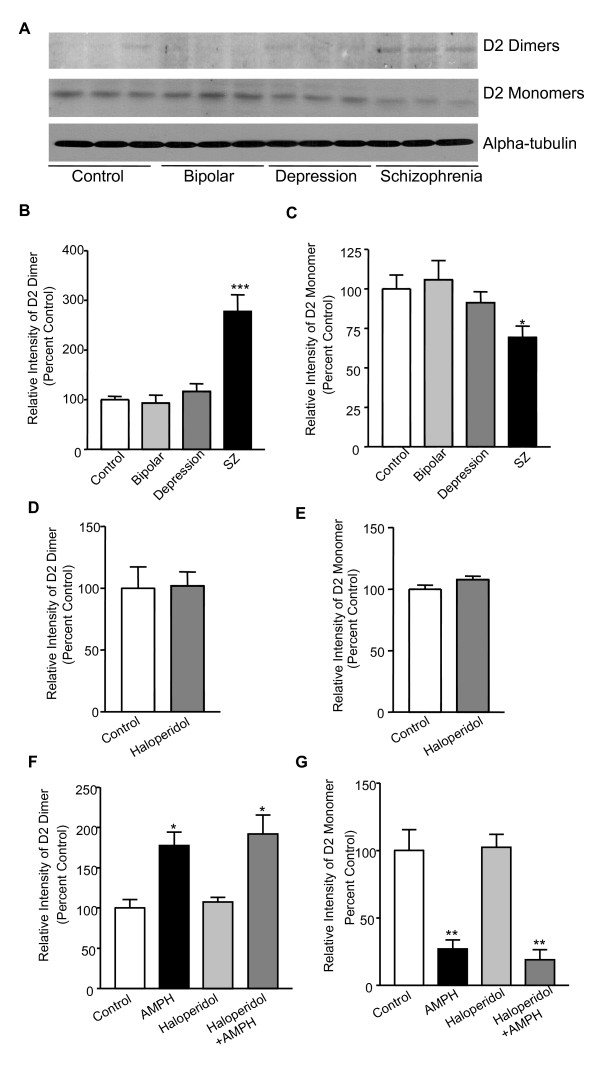
**D2R expression in postmortem striatal tissue of schizophrenia patients**. ***A. ***Representative Western blot analysis of the expression levels of D2R dimmers and monomers in human postmortem striatal sections of control, bipolar, depression and schizophrenia groups. α-tubulin was used as a loading control. ***B-C ***Bar graphs summarizing the Western blot data of D2R dimers (B) and monomers (C). *, ***Significantly different from control group (*p *< 0.05, *p *< 0.001; n = 15). Data were analyzed by One-way ANOVA, followed by SNK *post-hoc *test. ***D-E***, Chronic haloperidol treatment caused no significant changes in the expression of D2R dimers and monomers in rat striatal tissue compare to control group (n = 12). Data were analyzed by *t*-test. ***F-G***, Western blot analysis of the expression levels of D2R dimers (F) and monomers (G) in rat striatal slices treated with AMPH (30 min, 10 μM), in the presence or absence of haloperidol (30 min, 10 μM). *, **Significantly different from control group (*p *< 0.05, *p *< 0.01; n = 3). Data were analyzed by One-way ANOVA, followed by SNK *post-hoc *test.

As an initial step to investigate whether the expression pattern of D2R might be altered in schizophrenia, we carried out Western blot analysis with all 60 formalin-fixed paraffin-embedded human postmortem striatal sections from the Stanley Foundation, including 15 samples from each of four groups: control, bipolar disorder (BD), major depressive disorder (MDD) and schizophrenia (SZ). A polyclonal anti-D2 antibody that can specifically recognize both D2R dimers and monomers was used to examine the D2R expression in Western blot analysis (Additional File 1). As shown in Figure 1A-B, the expression level of D2R dimers exhibited a significant increase (277.7 ± 33.6%) in the postmortem striatal sections of schizophrenia patients (n = 15, *p *< 0.001), whereas the expression of D2R monomers showed a substantial decrease (69.3 ± 7.3%, Figure 1A, C). These data suggest D2R dimerization may contribute to the pathophysiology of schizophrenia.

We are aware of the fact that most of the schizophrenia patient samples (13/15) we examined were exposed to antipsychotic treatment ante-mortem. Thus, to rule out the possibility that the observed enhancement of D2R dimerization in the human postmortem schizophrenia striatal sections is the consequence of antipsychotic treatment, we tested the expression of D2R dimerization in striatal brain tissue of rats chronically treated with haloperidol. As shown in Figure 1D-E, haloperidol treatment failed to alter the degree of D2R dimerization. Furthermore, AMPH-induced D2R dimerization was not affected by the haloperidol treatment in rat striatal slices (Figure 1F-G). Taken together, these data suggest that the observed increment of D2R dimers may not be caused by the antipsychotic treatment.

### D2R dimerization was up-regulated in the striatal tissue of AISS rats

**Figure 2:**
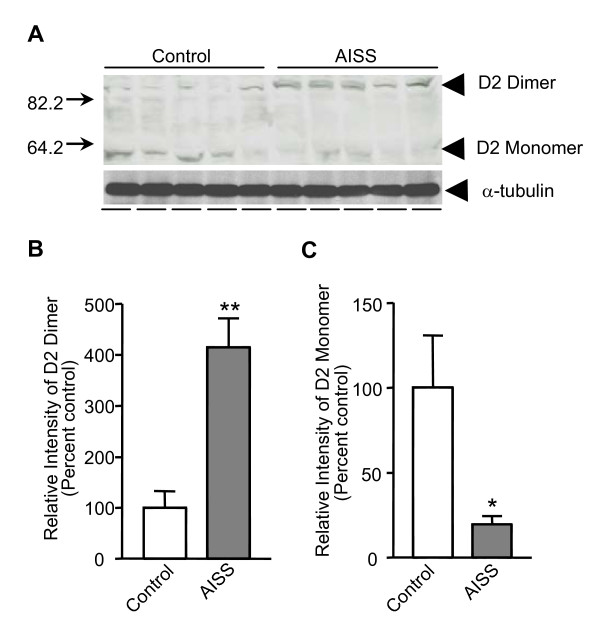
**Amphetamine induces D2R dimerization in AISS rat striatum**. ***A***, Western blot analysis of the expression levels of D2R dimers and monomers in striatal extracts of control and AISS rats. α-tubulin was used as a loading control. ***B-C***, Bar graphs summarizing the western blot data. *, **Significantly different from control group (*p *< 0.05, *p *< 0.01; n = 5). Data were analyzed by *t*-test.

Due to the similarity between schizophrenia and amphetamine psychosis [26-28], the amphetamine-induced sensitized state (AISS) has been widely used as an animal model of schizophrenia. Thus, we investigated whether the expression pattern of D2R is also altered in the striatum of AISS rats. As shown in Figure 2A-B, the expression of D2R dimer was significantly increased (415.3 ± 56.6%) in the AISS group compared to controls; the D2R monomer was significantly reduced in the AISS group compare to the control group (19.6 ± 5.0%, Figure 2A, C). These data suggest that chronic amphetamine exposure, which leads to a sensitized state and models many aspects of the clinical illness, also displays the same alteration that is seen in the postmortem striatal sections of schizophrenia patients.

### D2R dimerization may be associated with D2^high ^receptors

**Figure 3:**
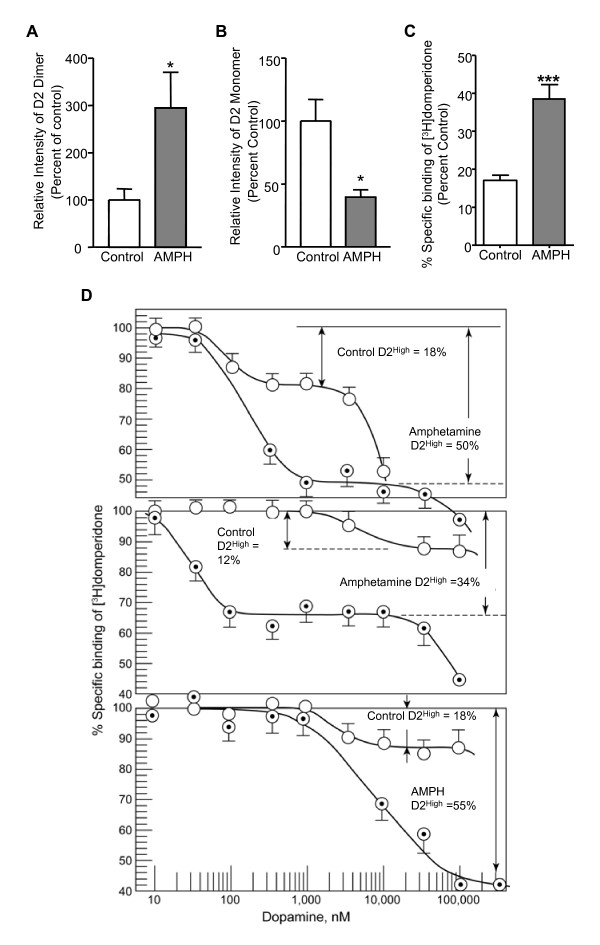
**Amphetamine facilitates D2R dimerization and the expression of high-affinity D2 receptors (D2**^**high**^**) in rat striatal brain slices**. ***A-B***, Western blot analysis of the expression levels of D2R dimers (A) and monomers (B) in rat striatal slices treated with or without AMPH (30 min, 10 μM). *Significantly different from control group (*p *< 0.05; n = 3). Data were analyzed by *t*-test. ***C***, Bar graph summarizing the binding of [^3^H]domperidone in rat striatal slices treated with or without AMPH (30 min, 10 μM). ***Significantly different from control group (*p *< 0.001; n = 7 in control group and n = 6 in AMPH group). ***D***, Graphs representing three samples out of six experiments. The competition between dopamine and [^3^H]domperidone showed a biphasic pattern. In control striata, low concentrations of dopamine (generally between 10 and 5,000 nM) inhibited the binding of [^3^H]domperidone by an average of 17 ± 1.4%. Higher concentrations of dopamine further reduced the binding of [^3^H]domperidone in a distinctly separate phase. There was a clear plateau between the high-affinity phase (i.e., at low concentrations of dopamine) and the low-affinity phase (i.e., at high concentrations of dopamine). The treatment with amphetamine increased the proportion of high-affinity D2 receptors to 38.8% ± 3.9%.

Previous studies have shown that in many animal models for psychosis, including the AISS, the proportion of D2R in the high-affinity state is elevated [29]. However, the molecular basis that renders D2R super-sensitive to dopamine remains unclear. As both D2R dimers and D2^high ^receptors are enhanced in the AISS animal model, we speculate that D2R dimerization may correlate with the D2R high-affinity state. We tested our hypothesis in rat striatal slices treated with amphetamine. Thus, rat striatal slices were treated with amphetamine and divided randomly into two groups: one for D2R dimer measurement and the other for D2^high ^receptors measurement. As shown in Figure 3A-B, acute amphetamine treatment significantly enhanced the expression of D2R dimers (295.0 ± 75.4%), while the D2R monomer expression was significantly decreased (39.9 ± 19.5%). Similarly, acute treatment with amphetamine increased the proportion of high-affinity D2R from 17 ± 1.4% to 38.8 ± 3.9% (Figure 3C), examples of which are shown in Figure 3D. These data suggest that D2R dimers may be associated with the super-sensitivity of D2R to dopamine.

### Amphetamine facilitates D2R dimerization in primary culture of rat striatal neurons

**Figure 4:**
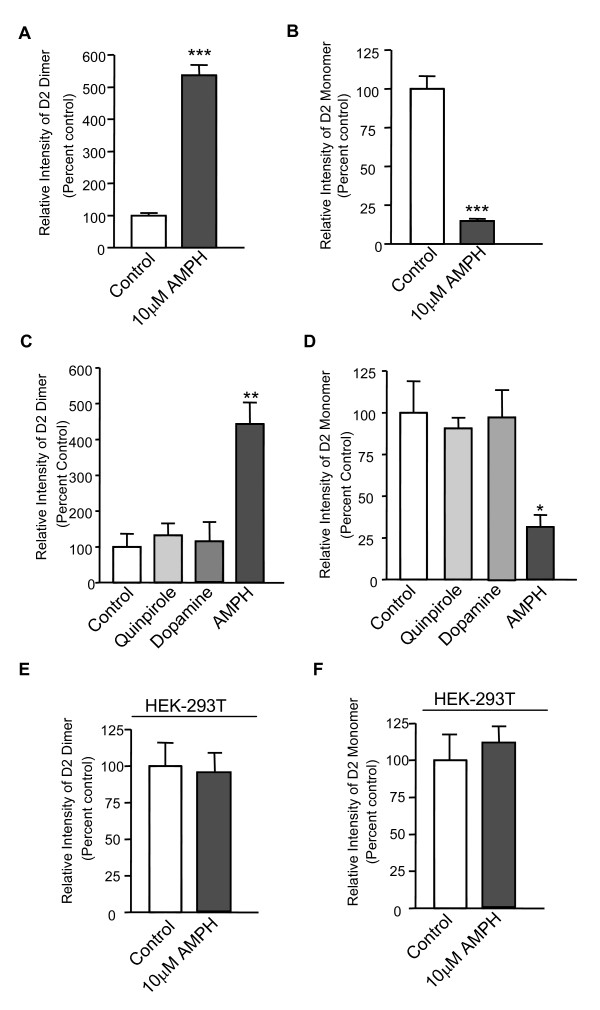
**Amphetamine facilitates D2R dimerization in rat striatal neurons**. ***A-B***, Western blot analysis of the expression levels of D2R dimers **(A) **and monomers **(B) **in primary cultures of rat striatal neurons treated with or without AMPH (30 min, 10 μM). ***Significantly different from control group (*p *< 0.001; n = 5). Data were analyzed by *t*-test. ***C-D***, Western blot analysis of the expression levels of D2R dimers **(C) **and monomers **(D) **in primary cultures of rat striatal neurons treated with quinpirole (10 μM), dopamine (10 μM) or AMPH (10 μM). *, **Significantly different from control group (*p *< 0.05, *p *< 0.01; n = 5). Data were analyzed by One-way ANOVA, followed by SNK *post-hoc *test. ***E-F***, Western blot analysis of the expression levels of D2R dimers **(E) **and monomers **(F) **in HEK-293T cells transfected with D2R in the presence or absence of AMPH (30 min, 10 μM; n = 3).

In order to identify the factors involving in the process of amphetamine-induced D2R dimerization, we then tested the amphetamine effect on D2R dimerization in a simplified experimental condition. Primary culture of rat striatal neurons was treated with 10 μM amphetamine for 30 min at 37°C. Consistent with the result from AISS rats, acute amphetamine treatment facilitated D2R dimerization (537.5 ± 37.5%, Figure 4A), whereas the expression of D2R monomers was significantly decreased (14.9 ± 1.4%) in amphetamine-treated groups (Figure 4B). Since amphetamine is able to enhance synaptic dopamine, we then tested whether the observed amphetamine-induced up-regulation of D2R dimerization is a consequence of activation of D2R. Both quinpirole (10 μM, 30 min), a specific D2R agonist, and dopamine (10 μM, 30 min) failed to up-regulate D2R dimerization (Figure 4C-D); indicating that D2R activation alone is not sufficient to account for the amphetamine-induced up-regulation of D2R dimerization. More interestingly, amphetamine stimulation also failed to up-regulate D2R dimerization in HEK-293T cells transfected only with D2R, suggesting the involvement of additional proteins that exist in striatal neurons but not in transfected cells (Figure 4E-F).

### D2R-DAT protein-protein interaction is involved in amphetamine-induced D2R dimerization

**Figure 5:**
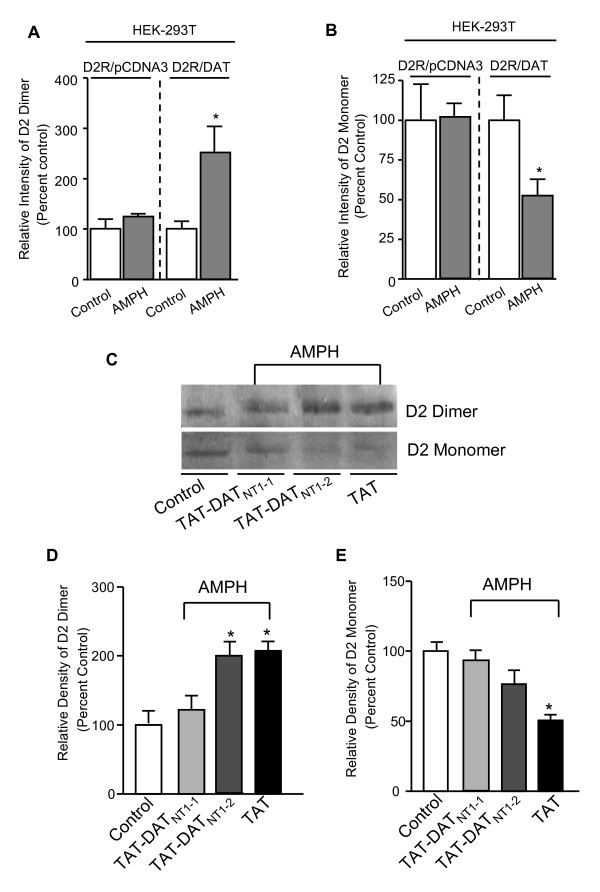
**Alteration of D2R-DAT protein-protein interaction in AISS rats**. ***A-B,***Western blot analysis of the expression levels of D2R dimers (A) and monomers (B) in HEK-293T cells co-expressing D2R/pcDNA3 or D2R/DAT in the presence or absence of AMPH (30 min, 10 μM; n = 3). *Significantly different from D2R/DAT control group (*p *< 0.05; n = 3). Data were analyzed by *t*-test. ***C***, Western blot analysis of the expression levels of D2R dimers and monomers in rat striatal slices treated with TAT-fused peptides (30 min, 10 μM) followed by amphetamine exposure (30 min, 10 μM). ***D-E***, Bar graphs summarizing the western blot data. *Significantly different from control group (*p *< 0.05, n = 3). Data were analyzed by One-way ANOVA, followed by SNK *post-hoc *test.

Previous studies have shown that amphetamine increases dopamine concentration in the synaptic cleft by reversing DAT-mediated dopamine uptake. Based on the fact that both D2R activation alone and amphetamine stimulation in HEK-293T cells expressing D2R failed to up-regulate D2R dimerization, we speculate that DAT may be one of the additional proteins that play a role in this process. To explore whether the existence of DAT is necessary for D2R dimerization, we examined the AMPH-induced D2R dimerization in HEK293T cells co-expressing D2R and DAT, and in HEK293T cells co-expressing D2R and pcDNA3, a mammalian expression vector in which DAT is subcloned. As shown in Figure5A-B, AMPH failed to up-regulate D2R dimerization in the absence of DAT. We have previously reported that the D2R forms a protein complex with DAT through direct protein-protein interactions [30]. Thus, if amphetamine induces up-regulation of D2R dimerization through a D2R-DAT interaction, disruption of the D2R-DAT interaction with the interfering peptide TAT-DAT_NT1-1 _encoding sequence of the interaction site of D2R-DAT within DAT [previously shown to disrupt D2R-DAT coupling [30]] should block the amphetamine-induced up-regulation of D2R dimerization. Consistent with our hypothesis, pre-treatment with TAT-DAT_NT1-1 _peptide (30 min, 10 μM) blocked amphetamine-induced enhancement of D2R dimer formation (Figure 5C-E), while TAT-only and TAT-DAT_NT1-2 _peptide (encodes scrambled sequence of TAT-DAT_NT1-1 _peptide) showed no effect. The ability of TAT-DAT_NT1-1 _peptide to disrupt D2R-DAT interactions was confirmed in a parallel co-immunoprecipitation experiment (data not shown). Taken together, these results suggest that D2R-DAT interaction may be necessary for the amphetamine-induced up-regulation of D2R dimerization and that this interaction may also contribute to the pathophysiology of schizophrenia.

## Discussion

In summary, we have identified a potential role for D2R dimerization in the pathology of schizophrenia. This conclusion is based on our observations of enhanced expression of D2R dimers in postmortem human striatal sections of schizophrenia patients and in the striatum of an animal model of schizophrenia (AISS), as well as the acute amphetamine-induced up-regulation of D2R dimerization. Additionally, we provided evidence that the physical coupling between D2R and DAT may be necessary for this amphetamine-induced up-regulation of D2R dimerization.

It has been long recognized that D2R exists in both monomers and dimers in brain. However, the mechanisms regulating this process as well as the physiological/pathological roles of dimerization remain unknown. Our data provide the first direct evidence that both acute and chronic amphetamine treatments regulate D2R dimerization. Previous studies have shown that amphetamine enhances synaptic dopamine concentrations by the blockade and reversal of DAT-mediated dopamine uptake [31-33]. Thus, an obvious explanation may be that the enhanced synaptic dopamine over-stimulates D2R and increases D2R dimerization. However, our results shown in Figure 4C-D indicate that activation of D2R is not sufficient to induce D2R dimerization. We also observed that acute amphetamine treatment failed to induce D2R dimerization in HEK-293T cells expressing D2R only (Figure 4E-F), suggesting the involvement of additional proteins/receptors in this process. Given that synaptic dopamine activates not only D2R but all dopamine receptor subtypes D1-D5, the observed amphetamine-induced up-regulation of D2R dimerization may also be involved in the activity of other dopamine receptors, such as D1R. Indeed, previous studies have shown a functional interaction between D1R and D2R, and the D1R and D2R may form a protein complex [34-36]. In addition to D1R, recent studies have shown that D2R can also interact with other receptors or channels, such as D3R [37], Somatostatin sst5 receptor [38], Adenosine A_2A _receptor [39] and Kir3 K^+ ^channel [40]. Several dopamine receptor interacting proteins (DRIPs) have also been identified to be directly and indirectly associated with D2R, including protein 4.1N/B/G [41], FilaminA [42], Spinophilin [43], GIPC [44], CAPS1 [45], ZIP [46], NCS-1 and GRK2 [47]. Future investigations may focus on studying the role of DRIPs as well as other D2R-interacting receptors in the AMPH-mediated D2R dimerization.

In many animal models of psychosis, the proportion of D2R in the high-affinity state is elevated 2- to 9-fold in the striatum of rats known to be supersensitive to dopamine [27,48,49]. Our current finding that amphetamine induces an increase in the proportion of D2^High ^receptors and an increase in the D2R dimers in rat tissue suggests that dimerization may be closely related to the dopamine-based hyperactivity elicited by amphetamine.

Previously, we reported that D2R directly interact with DAT, and the D2R-DAT protein complex formation enables D2R to up-regulate DAT-mediated dopamine uptake by recruiting DAT to the plasma membrane [30]. However, the role of D2R-DAT interaction in the regulation of D2R function remains unknown. In the current study, we provided evidence that disruption of the D2R-DAT interaction blocks amphetamine-induced up-regulation of D2R dimerization in transfected HEK293T cells, implicating the potential role of DAT in this process. However, it is worth noting that the AMPH-induced increase in D2R dimers seems to have bands that are more intense than the monomer bands. If the DAT is the only molecule required for this process, it may appear surprising that the DAT in such a small fraction of TH-positive neurons accounts for a large increase in DRD2 dimers in the cultured striatal neurons. Thus, there is a high probability that other additional molecules may be involved in D2R dimerization.

We have shown that D2R dimerization is enhanced in postmortem striatal sections from schizophrenia patients, and that amphetamine, a psychomotor stimulant that can induce psychosis, up-regulates D2R dimerization that in turn may be responsible for the noted dopamine hypersensitivity in schizophrenia. While the current antipsychotic treatments block dopamine D2 receptors, our data show that they do not alter the level of D2 dimers by themselves. Information from Stanley foundation indicated that 13 out of 15 patients in the schizophrenia group have received antipsychotic treatments and exhibited the increment of D2R dimerization. However, 12 out of 15 patients in the bipolar group have also received antipsychotic treatments but did not display an enhanced D2R dimerization. Therefore, a treatment focused at reversing the increased D2 dimerization my be closer to the pathophysiology of schizophrenia and may provide a novel therapeutic target for development of antipsychotics. Previous studies have shown that D2R forms dimers through multiple sites including transmembrane domain 4 [50]. Thus, further studies should be carried out to identify the exact interacting site(s) that are responsible for D2R dimer formation and interfering peptide(s) then can be developed and tested as potential antipsychotic agents in animal models of schizophrenia.

## Competing interests

There are no competing financial interests except for Dr. Shitij Kapur. Dr. Shitij Kapur declares that he has financial association (Grant Support) with *AstraZeneca, Bristol-Myers Squibb (BMS) and Glaxo Smith Kline *(listed alphabetically) over the past three years.

## Authors' contributions

MW carried out all experiments, with the exception of the preparation of acute striatal slices and the [^3^H]-dopamine binding assay. LP helped to prepare the acute rat striatal slices. PJF and SK provided brain tissue from AISS animal model and helped to edit the manuscript. PS conducted the [^3^H]-dopamine binding assay and helped to edit the manuscript. FL supervised the study and wrote the manuscript. All authors read and approved the final manuscript.

## Supplementary Material

Additional file 1 **Specificity of D2R antibody**. Western blot analysis of dopamine receptor D2 dimer and monomer expression in both rat and human striatal extracts. Click here for file

## References

[B1] Frankle WG Laruelle M Neuroreceptor imaging in psychiatric disorders Ann Nucl Med 2002 16 437 446 10.1007/BF02988639 12508833

[B2] Seeman P Kapur S Schizophrenia: more dopamine, more D2 receptors Proc Natl Acad Sci USA 2000 97 7673 7675 10.1073/pnas.97.14.7673 10884398 PMC33999

[B3] Hirvonen J van Erp TG Huttunen J Aalto S Nagren K Huttunen M Lonnqvist J Kaprio J Hietala J Cannon TD Increased caudate dopamine D2 receptor availability as a genetic marker for schizophrenia Arch Gen Psychiatry 2005 62 371 378 10.1001/archpsyc.62.4.371 15809404

[B4] Howes OD Kapur S The dopamine hypothesis of schizophrenia: version III--the final common pathway Schizophr Bull 2009 35 549 562 10.1093/schbul/sbp006 19325164 PMC2669582

[B5] George SR O'Dowd BF Lee SP G-protein-coupled receptor oligomerization and its potential for drug discovery Nat Rev Drug Discov 2002 1 808 820 10.1038/nrd913 12360258

[B6] Sartania N Appelbe S Pediani JD Milligan G Agonist occupancy of a single monomeric element is sufficient to cause internalization of the dimeric beta2-adrenoceptor Cell Signal 2007 19 1928 1938 10.1016/j.cellsig.2007.05.002 17561373

[B7] Parenty G Appelbe S Milligan G CXCR2 chemokine receptor antagonism enhances DOP opioid receptor function via allosteric regulation of the CXCR2-DOP receptor heterodimer Biochem J 2008 412 245 256 10.1042/BJ20071689 18307412 PMC2474558

[B8] Vilardaga JP Nikolaev VO Lorenz K Ferrandon S Zhuang Z Lohse MJ Conformational cross-talk between alpha2A-adrenergic and mu-opioid receptors controls cell signaling Nat Chem Biol 2008 4 126 131 10.1038/nchembio.64 18193048

[B9] Zylbergold P Hebert TE A division of labor: asymmetric roles for GPCR subunits in receptor dimers Nat Chem Biol 2009 5 608 609 10.1038/nchembio0909-608 19690532

[B10] Rives ML Vol C Fukazawa Y Tinel N Trinquet E Ayoub MA Shigemoto R Pin JP Prezeau L Crosstalk between GABA(B) and mGlu1a receptors reveals new insight into GPCR signal integration EMBO J 2009 28 2195 2208 10.1038/emboj.2009.177 19590495 PMC2726695

[B11] Fuxe K Agnati LF Benfenati F Celani M Zini I Zoli M Mutt V Evidence for the existence of receptor--receptor interactions in the central nervous system. Studies on the regulation of monoamine receptors by neuropeptides J Neural Transm Suppl 1983 18 165 179 6192208

[B12] Ng GY O'Dowd BF Caron M Dennis M Brann MR George SR Phosphorylation and palmitoylation of the human D2L dopamine receptor in Sf9 cells J Neurochem 1994 63 1589 1595 10.1046/j.1471-4159.1994.63051589.x 7931316

[B13] Ng GY O'Dowd BF Lee SP Chung HT Brann MR Seeman P George SR Dopamine D2 receptor dimers and receptor-blocking peptides Biochem Biophys Res Commun 1996 227 200 204 10.1006/bbrc.1996.1489 8858125

[B14] Zawarynski P Tallerico T Seeman P Lee SP O'Dowd BF George SR Dopamine D2 receptor dimers in human and rat brain FEBS Lett 1998 441 383 386 10.1016/S0014-5793(98)01588-9 9891976

[B15] Franco R Neurotransmitter receptor heteromers in neurodegenerative diseases and neural plasticity J Neural Transm 2009 116 983 987 10.1007/s00702-008-0148-y 19002553

[B16] Fuxe K Marcellino D Rivera A Diaz-Cabiale Z Filip M Gago B Roberts DC Langel U Genedani S Ferraro L Receptor-receptor interactions within receptor mosaics. Impact on neuropsychopharmacology Brain Res Rev 2008 58 415 452 10.1016/j.brainresrev.2007.11.007 18222544

[B17] Armstrong D Strange PG Dopamine D2 receptor dimer formation: evidence from ligand binding J Biol Chem 2001 276 22621 22629 10.1074/jbc.M006936200 11278324

[B18] Seeman P Guan HC Civelli O Van Tol HH Sunahara RK Niznik HB The cloned dopamine D2 receptor reveals different densities for dopamine receptor antagonist ligands. Implications for human brain positron emission tomography Eur J Pharmacol 1992 227 139 146 10.1016/0922-4106(92)90121-B 1358662

[B19] Torrey EF Webster M Knable M Johnston N Yolken RH The stanley foundation brain collection and neuropathology consortium Schizophr Res 2000 44 151 155 10.1016/S0920-9964(99)00192-9 10913747

[B20] Turrone P Remington G Kapur S Nobrega JN Differential effects of within-day continuous vs. transient dopamine D2 receptor occupancy in the development of vacuous chewing movements (VCMs) in rats Neuropsychopharmacology 2003 28 1433 1439 10.1038/sj.npp.1300233 12838271

[B21] Liu F Wan Q Pristupa ZB Yu XM Wang YT Niznik HB Direct protein-protein coupling enables cross-talk between dopamine D5 and gamma-aminobutyric acid A receptors Nature 2000 403 274 280 10.1038/35001232 10659839

[B22] Lee FJ Xue S Pei L Vukusic B Chery N Wang Y Wang YT Niznik HB Yu XM Liu F Dual regulation of NMDA receptor functions by direct protein-protein interactions with the dopamine D1 receptor Cell 2002 111 219 230 10.1016/S0092-8674(02)00962-5 12408866

[B23] Seeman P Ulpian C Wreggett KA Wells JW Dopamine receptor parameters detected by [3H]spiperone depend on tissue concentration: analysis and examples J Neurochem 1984 43 221 235 10.1111/j.1471-4159.1984.tb06700.x 6726248

[B24] Seeman P Tallerico T Ko F Dopamine displaces [3H]domperidone from high-affinity sites of the dopamine D2 receptor, but not [3H]raclopride or [3H]spiperone in isotonic medium: Implications for human positron emission tomography Synapse 2003 49 209 215 10.1002/syn.10232 12827639

[B25] Aarts M Liu Y Liu L Besshoh S Arundine M Gurd JW Wang YT Salter MW Tymianski M Treatment of ischemic brain damage by perturbing NMDA receptor-PSD-95 protein interactions Science 2002 298 846 850 10.1126/science.1072873 12399596

[B26] Featherstone RE Kapur S Fletcher PJ The amphetamine-induced sensitized state as a model of schizophrenia Prog Neuropsychopharmacol Biol Psychiatry 2007 31 1556 1571 10.1016/j.pnpbp.2007.08.025 17884274

[B27] Seeman P Schwarz J Chen JF Szechtman H Perreault M McKnight GS Roder JC Quirion R Boksa P Srivastava LK Psychosis pathways converge via D2high dopamine receptors Synapse 2006 60 319 346 10.1002/syn.20303 16786561

[B28] Ujike H Sato M Clinical features of sensitization to methamphetamine observed in patients with methamphetamine dependence and psychosis Ann N Y Acad Sci 2004 1025 279 287 10.1196/annals.1316.035 15542728

[B29] Seeman P McCormick PN Kapur S Increased dopamine D2(High) receptors in amphetamine-sensitized rats, measured by the agonist [(3)H](+)PHNO Synapse 2007 61 263 267 10.1002/syn.20367 17318886

[B30] Lee FJ Pei L Moszczynska A Vukusic B Fletcher PJ Liu F Dopamine transporter cell surface localization facilitated by a direct interaction with the dopamine D2 receptor EMBO J 2007 26 2127 2136 10.1038/sj.emboj.7601656 17380124 PMC1852782

[B31] Heikkila RE Orlansky H Cohen G Studies on the distinction between uptake inhibition and release of (3H)dopamine in rat brain tissue slices Biochem Pharmacol 1975 24 847 852 10.1016/0006-2952(75)90152-5 1125084

[B32] Jones SR Gainetdinov RR Wightman RM Caron MG Mechanisms of amphetamine action revealed in mice lacking the dopamine transporter J Neurosci 1998 18 1979 1986 9482784 10.1523/JNEUROSCI.18-06-01979.1998 PMC6792915

[B33] Seiden LS Sabol KE Ricaurte GA Amphetamine: effects on catecholamine systems and behavior Annu Rev Pharmacol Toxicol 1993 33 639 677 10.1146/annurev.pa.33.040193.003231 8494354

[B34] Free RB Hazelwood LA Cabrera DM Spalding HN Namkung Y Rankin ML Sibley DR D1 and D2 dopamine receptor expression is regulated by direct interaction with the chaperone protein calnexin J Biol Chem 2007 282 21285 21300 10.1074/jbc.M701555200 17395585

[B35] Lee SP So CH Rashid AJ Varghese G Cheng R Lanca AJ O'Dowd BF George SR Dopamine D1 and D2 receptor Co-activation generates a novel phospholipase C-mediated calcium signal J Biol Chem 2004 279 35671 35678 10.1074/jbc.M401923200 15159403

[B36] Rashid AJ So CH Kong MM Furtak T El-Ghundi M Cheng R O'Dowd BF George SR D1-D2 dopamine receptor heterooligomers with unique pharmacology are coupled to rapid activation of Gq/11 in the striatum Proc Natl Acad Sci USA 2007 104 654 659 10.1073/pnas.0604049104 17194762 PMC1766439

[B37] Scarselli M Novi F Schallmach E Lin R Baragli A Colzi A Griffon N Corsini GU Sokoloff P Levenson R D2/D3 dopamine receptor heterodimers exhibit unique functional properties J Biol Chem 2001 276 30308 30314 10.1074/jbc.M102297200 11373283

[B38] Rocheville M Lange DC Kumar U Patel SC Patel RC Patel YC Receptors for dopamine and somatostatin: formation of hetero-oligomers with enhanced functional activity Science 2000 288 154 157 10.1126/science.288.5463.154 10753124

[B39] Hillion J Canals M Torvinen M Casado V Scott R Terasmaa A Hansson A Watson S Olah ME Mallol J Coaggregation, cointernalization, and codesensitization of adenosine A2A receptors and dopamine D2 receptors J Biol Chem 2002 277 18091 18097 10.1074/jbc.M107731200 11872740

[B40] Lavine N Ethier N Oak JN Pei L Liu F Trieu P Rebois RV Bouvier M Hebert TE Van Tol HH G protein-coupled receptors form stable complexes with inwardly rectifying potassium channels and adenylyl cyclase J Biol Chem 2002 277 46010 46019 10.1074/jbc.M205035200 12297500

[B41] Binda AV Kabbani N Lin R Levenson R D2 and D3 dopamine receptor cell surface localization mediated by interaction with protein 4.1N Mol Pharmacol 2002 62 507 513 10.1124/mol.62.3.507 12181426

[B42] Li M Bermak JC Wang ZW Zhou QY Modulation of dopamine D(2) receptor signaling by actin-binding protein (ABP-280) Mol Pharmacol 2000 57 446 452 10692483 10.1124/mol.57.3.446

[B43] Smith FD Oxford GS Milgram SL Association of the D2 dopamine receptor third cytoplasmic loop with spinophilin, a protein phosphatase-1-interacting protein J Biol Chem 1999 274 19894 19900 10.1074/jbc.274.28.19894 10391935

[B44] Jeanneteau F Diaz J Sokoloff P Griffon N Interactions of GIPC with dopamine D2, D3 but not D4 receptors define a novel mode of regulation of G protein-coupled receptors Mol Biol Cell 2004 15 696 705 10.1091/mbc.E03-05-0293 14617818 PMC329290

[B45] Binda AV Kabbani N Levenson R Regulation of dense core vesicle release from PC12 cells by interaction between the D2 dopamine receptor and calcium-dependent activator protein for secretion (CAPS) Biochem Pharmacol 2005 69 1451 1461 10.1016/j.bcp.2005.02.015 15857609

[B46] Kim OJ Ariano MA Namkung Y Marinec P Kim E Han J Sibley DR D2 dopamine receptor expression and trafficking is regulated through direct interactions with ZIP J Neurochem 2008 106 83 95 10.1111/j.1471-4159.2008.05348.x 18346199 PMC5095932

[B47] Kabbani N Negyessy L Lin R Goldman-Rakic P Levenson R Interaction with neuronal calcium sensor NCS-1 mediates desensitization of the D2 dopamine receptor J Neurosci 2002 22 8476 8486 12351722 10.1523/JNEUROSCI.22-19-08476.2002 PMC6757796

[B48] Seeman P Schizophrenia model of elevated D2(High) receptors: haloperidol reverses the amphetamine-induced elevation in dopamine D2(High) Schizophr Res 2009 109 191 192 10.1016/j.schres.2008.12.024 19171464

[B49] Seeman P Weinshenker D Quirion R Srivastava LK Bhardwaj SK Grandy DK Premont RT Sotnikova TD Boksa P El-Ghundi M Dopamine supersensitivity correlates with D2High states, implying many paths to psychosis Proc Natl Acad Sci USA 2005 102 3513 3518 10.1073/pnas.0409766102 15716360 PMC548961

[B50] Lee SP O'Dowd BF Rajaram RD Nguyen T George SR D2 dopamine receptor homodimerization is mediated by multiple sites of interaction, including an intermolecular interaction involving transmembrane domain 4 Biochemistry 2003 42 11023 11031 10.1021/bi0345539 12974638

