# Supplementary material for: Schizophrenia, amphetamine-induced sensitized state and acute amphetamine exposure all show a common alteration: increased dopamine D2 receptor dimerization
Source: Mol Brain. 2010 Sep 2;3:25. doi: 10.1186/1756-6606-3-25 (PMC2942879; doi:10.1186/1756-6606-3-25)
Supplement: Additional file 1 — Specificity of D2R antibody. Western blot analysis of dopamine receptor D2 dimer and monomer expression in both rat and human striatal extracts. [file 1756-6606-3-25-S1.PDF]

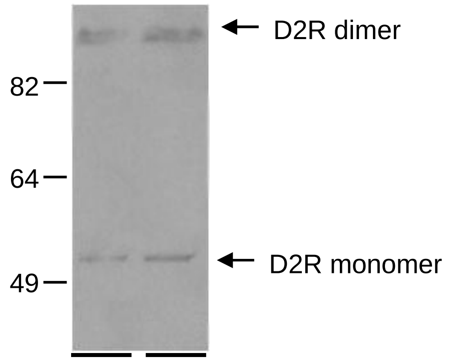


**Additional File 1. Specificity of D2R antibody.** Western blot analysis of dopamine receptor D2 dimer and monomer expression in rat striatal extracts (left lane) and in human striatal extracts (right lane).
